# Necroptosis in anti-viral inflammation

**DOI:** 10.1038/s41418-018-0172-x

**Published:** 2018-07-26

**Authors:** Himani Nailwal, Francis Ka-Ming Chan

**Affiliations:** Department of Pathology, Immunology and Microbiology Program, University of Massachusetts Medical School, 368 Plantation Street, Worcester, MA 01605 USA

**Keywords:** Cell death and immune response, Inflammation

## Abstract

The primary function of the immune system is to protect the host from invading pathogens. In response, microbial pathogens have developed various strategies to evade detection and destruction by the immune system. This tug-of-war between the host and the pathogen is a powerful force that shapes organismal evolution. Regulated cell death (RCD) is a host response that limits the reservoir for intracellular pathogens such as viruses. Since pathogen-specific T cell and B cell responses typically take several days and is therefore slow-developing, RCD of infected cells during the first few days of the infection is critical for organismal survival. This innate immune response not only restricts viral replication, but also serves to promote anti-viral inflammation through cell death-associated release of damage-associated molecular patterns (DAMPs). In recent years, necroptosis has been recognized as an important response against many viruses. The central adaptor for necroptosis, RIPK3, also exerts anti-viral effects through cell death-independent activities such as promoting cytokine gene expression. Here, we will discuss recent advances on how viruses counteract this host defense mechanism and the effect of necroptosis on the anti-viral inflammatory reaction.

## Abstract (web summary)

Necroptosis facilitates anti-viral inflammation, which is countered by virally-encoded inhibitors.

## Graphical abstract

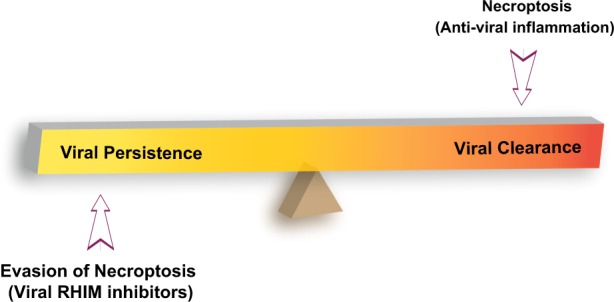

## Facts

- Necroptosis is mediated through cellular RHIM domain-containing proteins, RIPK1, RIPK3, ZBP1, and TRIF.
- Virus infections activate RHIM-dependent necroptosis in host, which leads to anti-viral inflammation.
- Viruses modulate necroptosis pathway through various inhibitors competing for RHIM binding.
- RIPK3 activates the NLRP3 inflammasome in certain RNA virus infection independent of its necroptotic function.

## Open questions

- Is necroptosis a primary driver or secondary form of cell death upon virus infection?
- Do lytic viruses like IAV activate necroptosis contribute to pathogenesis?
- Is ZBP1 sensing of viral RNA a common phenomenon among different virus infections?
- Does RIPK3 have roles beyond necroptosis in virus infections?
- Can targeting cellular RHIM proteins be an effective strategy as anti-viral therapeutics?

## Introduction

Regulated cell death (RCD) constitutes an important aspect of organismal development. RCD drives crucial cellular responses and is especially important for immune homeostasis. Until recently, apoptosis was synonymous with programmed cell death. Apoptosis is characterized by cell shrinking, chromatin condensation, nuclear fragmentation (karyorrhexis) and plasma membrane blebbing. Apoptotic cells also expose the “eat-me” signal phosphatidyl serine on the cell surface to promote engulfment by resident phagocytes. Thus, apoptosis can be considered a “contained” and immunologically silent form of cell death. This is in contrast to cell death by necrosis, which is marked by cell lysis and the release of inflammatory mediators. Although non-apoptotic cell death pathways are sometimes considered as secondary responses to apoptosis, there is growing evidence that they can also be “programmed” and controlled by a dedicated molecular circuitry. Moreover, these pathways can be activated independently or as a consequence of apoptosis inhibition [1].

Many forms of non-apoptotic RCD have been described in recent years. These include necroptosis, parthanatos, ferroptosis, mitochondrial permeability transition (MPT)-dependent necrosis, pyroptosis and pyronecrosis, and NETosis or ETosis [1]. The molecular mechanisms and biological roles for some of these cell death modes are still poorly defined. By contrast, necroptosis has emerged as a pivotal cell death response in microbial infections. Necroptosis is initiated in response to toll-like receptor 3 (TLR3), TLR4, and death receptors in the tumor necrosis factor receptor (TNFR) superfamily. Necroptosis is marked by rupture of the plasma membrane and release of pro-inflammatory damage-associated molecular patterns (DAMPs) (Box 1). Hence, necroptosis is distinct from apoptosis in both morphology and biological consequences [2]. Receptor interacting protein kinase 3 (RIPK3) is a serine/threonine kinase and key adaptor of necroptosis. In addition to the kinase domain, RIPK3 also contains a “RIP homotypic interaction motif (RHIM)” at the C-terminus. As its name implies, the RHIM of RIPK3 mediates homotypic interaction with other mammalian RHIM-containing signal adaptors [3]. Consistent with the pro-inflammatory role of necroptosis, RIPK3 has critical functions in microbial infections [4]. Here, we discuss how viruses target RIPK3 and other components of the necroptosis pathway to manipulate host-defense and anti-viral inflammation (Table 1).

**Table1:** Summary of virus-driven modulation of necroptosis

Virus	Host species	Necroptosis pathway inhibition or activation	Virus encoded Necroptosis inhibitor or inducer	Mechanism of necroptosis modulation
VV	Murine	Inhibition of Type I IFN-induced necroptosis	E3L	E3L binding to the DNA binding (Zα2) domain of ZBP1 to disrupt viral RNA sensing by ZBP1 [12]
MCMV	Murine	Inhibition of ZBP1/RIPK3-mediated necroptosis	M45/vIRA	Disruption of RHIM–RHIM interaction between ZBP1 and RIPK3 [16, 17]
HSV-1	Human	Inhibition of ZBP1/RIPK3-mediated necroptosis	ICP6	Disrupting RHIM–RHIM interaction between ZBP1 and RIPK3 [27, 28]
HSV-2	Human	Inhibition of ZBP1/RIPK3-mediated necroptosis	ICP10	Disrupting RHIM–RHIM interaction between ZBP1 and RIPK3 [29]
EBV	Human	Inhibition of TNFα induced necroptosis	LMP1	Modulating the ubiquitination status of RIPK1 and RIPK3 [30]
HCMV	Human	Inhibition of RIPK3-mediated necroptosis	Undetermined	Undetermined
IAV	Human	Inhibition of ZBP1/RIPK3-mediated necroptosis	Undetermined	ZBP1 sensing of viral RNA and/or the viral proteins NP and PB1 [43, 44]
Sendai virus	Murine	Activation of RIPK3-mediated necroptosis in the presence of zVAD-fmk	Undetermined	RIG-I sensing of Sendai virus RNA [41]

**Fig. 1:**
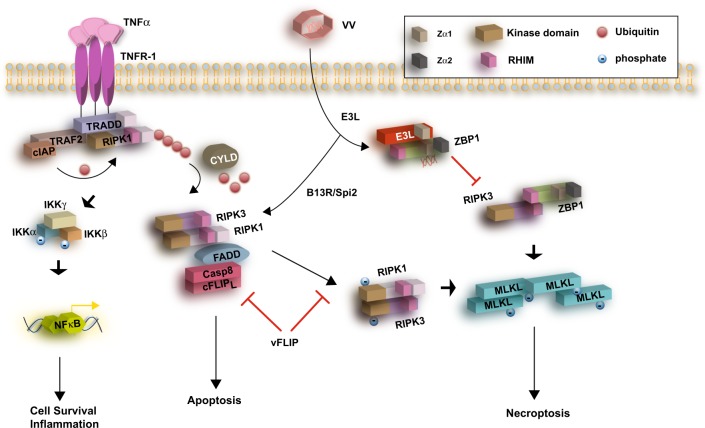
Necroptosis in poxvirus infection. Control of vaccinia virus (VV) infection requires TNF signaling [9]. TNF signaling through its trimeric receptor (TNFR-1) triggers three distinct signaling responses: NF-κB activation, apoptosis or necroptosis. Vaccinia virus encodes the caspase inhibitor B13R/Spi2, which inhibits caspase 1 and caspase 8 and blocks apoptosis. Thus, in VV infection, TNF stimulation favors necroptosis. This leads to assembly of the RHIM–RHIM interaction between RIPK1–RIPK3 and necrosome formation. Certain viral FLIPs such as MC159 from *Molluscum contagious* virus (MCV) inhibit both apoptosis and necroptosis, although the mechanisms are not fully elucidated. The VV-encoded protein E3L contains a z-DNA binding domain that interacts with ZBP1 and senses viral RNA. E3L binding sequesters ZBP1 from RIPK3 and therefore prevents necroptosis. However, E3L does not interfere with TNF-induced necroptosis. VV Vaccinia virus, TRADD TNFRSF1A associated death domain, TRAF2 TNF receptor associated factor 2, cIAP cellular inhibitor of apoptosis, CYLD cylindromatosis, FADD Fas associated via death domain, IKK inhibitor of nuclear factor kappa B kinase, cFLIPL CASP8 and FADD like apoptosis regulator long isoform, MLKL mixed lineage kinase domain-like, RHIM RIP homotypic interaction motif, Zα1, Zα2 zDNA binding domain

Box 1 Necroptosis signaling pathway Although stimulated by several death ligands of the Tumor necrosis factor (TNF) superfamily (TNFR1, Fas/CD95 and TRAIL-R) and Toll-like receptors, TLR3 and TLR4, most of our understanding of necroptosis comes from the studies of TNFα/TNFR1 pathway. One of the key effects of TNFα/TNFR1 pathway is NF-κB transcriptional activation and its subsequent pro-survival effects. The NF-κB activation takes place in the plasma membrane-associated “Complex I”, which is assembled upon death domain-mediated recruitment of TNFR1-associated Death domain adaptor protein (TRADD), and receptor interacting protein kinase 1 (RIPK1) (Fig. 1) [67]. Complex I assembly serves as a central checkpoint for the induction of NF-κB, apoptosis and necroptosis. E3 ligases such as cIAP1/2 and LUBAC mediate K63 and linear ubiquitination of RIPK1, ultimately resulting in IκBα phosphorylation by IKKα/β and IκBα degradation. This liberates NF-κB for nuclear translocation and transcription of inflammatory effectors. The apoptotic function of TNFα signaling is mediated through Fas-associated death domain (FADD) and pro-caspase 8 in the cytoplasmic complex II [67]. The switch from complex I to complex II is driven by the deubiquitination of RIPK1 by the deubiquitinating enzyme CYLD (Fig. 1) [68]. Furthermore, in the presence of caspase-8 inhibition, as seen in certain virus infections [69], complex IIb, which is also known as the “necrosome”, is formed. This complex is marked by the interaction of RIPK1 and RIPK3 through their respective RHIM domains. This RHIM-mediated interaction is marked by filamentous β-amyloid-like conversion of the complex, which promotes robust necroptosis signaling [70].

The execution of necroptosis requires RIPK3-induced phosphorylation of mixed lineage kinase domain-like (MLKL) at Thr357 and Ser358 [71]. MLKL phosphorylation stimulates its oligomerization, membrane translocation and rupture of the cell membrane [72–74]. Several models have been proposed to explain the membrane rupture activation of MLKL. These include the four α-helix bundle (4HB) domain of MLKL promoting Ca^2+^ influx through TRPM7 channels [73] and MLKL contacting certain phospholipids in the plasma membrane to form membrane-penetrating pores [74].

## Necroptosis upon poxvirus infection

RIPK1, the upstream activator of RIPK3 in the TNF receptor pathway, is a cleavage substrate of caspase 8. Caspase 8 cleaves RIPK1 at D324, the boundary between the N-terminal kinase domain and the C-terminal RHIM and death domain [5]. The C-terminal cleavage product, which contains the death domain, was reported to enhance apoptosis by promoting TRADD and FADD interaction [5]. However, since this cleavage event results in the release of the kinase domain from the signaling complex, necroptosis is inhibited. This explains why inhibition of caspase 8 is a key priming signal for necroptosis. Although the supporting biochemical evidence is still lacking, similar cleavage of RIPK3 by caspase 8 has also been implicated [6, 7]. Large DNA viruses often target caspases for inhibition, a strategy that promotes viral persistence and immune evasion within the host. For example, poxviruses encode serpins that inhibit caspase 1 and caspase 8 [8]. Poxviral inhibition of caspase 8 may therefore provide a natural priming signal for necroptosis. Indeed, cells infected with vaccinia virus (VV), the poxvirus strain that is used to vaccinate the world population against smallpox, are highly sensitive to TNF-induced necroptosis [9]. By contrast, VV-infected cells deficient in RIPK1 or RIPK3 were resistant to TNF-induced necroptosis [10]. As a consequence, *Ripk3*^−/−^ mice and mice expressing the kinase-inactive RIPK1 mutant D138N both failed to control VV replication in vivo [10, 11]. The phenotypes of *Ripk3*^−/−^ mice were reminiscent of mice lacking TNF or TNF receptor expression [9]. These results indicate that TNF, RIPK1, and RIPK3-dependent necroptosis is a key innate immune host defense mechanism against VV.

Interferon (IFN) response is a powerful host response against pathogens. VV encodes many immune evasion genes including those that neutralize the inflammatory cytokines TNF and IFNs and other innate immune signaling pathways. One of the VV-encoded immune modulators is E3L, which contains a z-DNA binding domain that is also found in the mammalian RHIM adaptor z-DNA binding protein 1 (ZBP1, aka DAI for DNA activator of interferon). Infection with VV expressing a truncated E3L lacking this z-DNA binding domain led to increased ZBP1 expression and excessive Type I IFN-induced RIPK3/MLKL-dependent necroptosis. Moreover, the E3L mutant VV failed to cause disease in wild type mice [12]. Strikingly, pathogenicity was restored when the E3L mutant VV was used to infect *Ripk3*^−/−^ and *Zbp1*^−/−^ mice [12]. These results indicate that while TNF-induced, RIPK1/RIPK3/MLKL-mediated necroptosis confers protection to the host, E3L-mediated inhibition of ZBP1 and RIPK3 enables VV to evade the anti-viral effects of IFNs (Fig. 1). The opposing effects of the two RIPK3-driven responses suggest that the timing and context of RIPK3-mediated necroptosis may impact host response as well as viral growth and dissemination in the host.

## Necroptosis upon herpesvirus infection

### Cytomegalovirus

Herpesvirus is another large DNA virus family that exploits the necroptosis pathway. In a forward genetic screen using transposon mutagenesis, Brune and colleagues identified the murine cytomegalovirus (MCMV) encoded M45 gene as a factor that promotes viral growth and endothelial cell survival [13]. M45 encodes a large polypeptide with homology to the large subunit of ribonucleotide reductase (R1) at the C-terminus. However, unlike other viral R1 subunits, M45 lacks enzymatic activity [14]. In addition to the R1-like domain, M45 contains a RHIM at the N-terminus that mediates binding to mammalian RHIM-containing adaptors such as RIPK1 and RIPK3 [15, 16]. In over-expression studies, M45 inhibited both TNF- and TLR3-driven NF-κB and MAPK activation [15]. More recent studies showed that M45 also inhibits necroptosis during MCMV infection. For example, mutant MCMV expressing a M45 RHIM mutant failed to replicate in cells due to excessive activation of RIPK3-dependent necroptosis [17]. However, M45 was only required for optimal viral replication in certain cell types in tissue culture [18]. Mutant virus replication was also compromised in the lungs, spleen, and salivary glands of wild type mice. Thus, M45 is the first viral inhibitor of RIP activation (vIRA) identified. Strikingly, replication of the M45 RHIM mutant virus was restored in *Ripk3*^−/−^ or *Zbp1*^−/−^ cells and mice [19], indicating that M45 prevents premature cell death by inhibiting ZBP1/RIPK3-induced necroptosis. In contrast to TNF-induced necroptosis in VV-infected cells, RIPK1 is dispensable for necroptosis induced by the M45 RHIM mutant MCMV [17]. These results establish necroptosis as a bona fide host defense mechanism in MCMV. They also provide the first examples in which mammalian RHIM-containing signal adaptors, other than RIPK1, activate RIPK3 and necroptosis.

Recent studies suggest that MCMV transcription is required for ZBP1-dependent necroptosis. When MCMV transcription is inhibited at an early time point with chemical inhibitors, M45 RHIM mutant virus-infected cells become resistant to necroptosis. The viral immediate early protein 3 (IE3), a crucial transcriptional activator of MCMV, was required for ZBP1 activation. Moreover, mutation of key amino acid residues in the z-DNA binding domain of ZBP1 abolished this response, suggesting that ZBP1 activates necroptosis upon sensing viral nucleic acid [20]. Hence, viral RNA, but not viral DNA, appears to be the ligand that activates ZBP1 in response to MCMV infection [21].

**Fig. 2:**
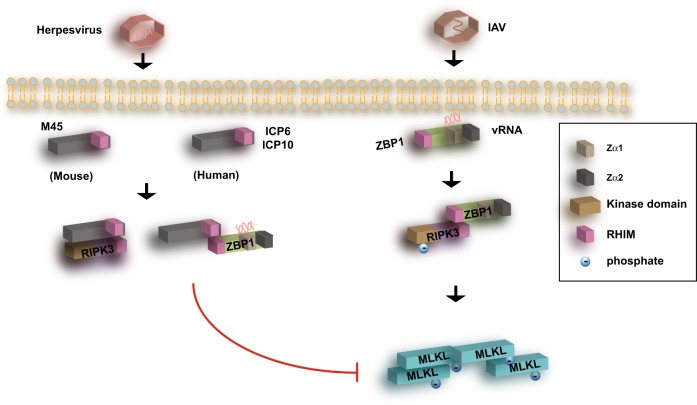
Necroptosis in herpesvirus and Influenza virus infection. Herpesviruses exploit the necroptotic pathway via various RHIM adaptors such as M45 from MCMV, ICP6 from HSV-1 and ICP10 from HSV-2. These viral inhibitors sequester ZBP1 and RIPK3 to prevent their interaction and activation. When viral RHIM inhibitors are absent, ZBP1 senses viral RNA (vRNA) to trigger RIPK3 binding and activation. The necroptosis modulating function of these viral RHIM adaptors varies with host species. For example, ICP6 and ICP10 prevent necroptosis in human cells, but stimulate RIPK3-dependent necroptosis in mouse cells. Though the molecular basis for the differential effects of ICP6 or ICP10 in different species is unknown, it may account for the species tropism of these viruses. In contrast to herpesvirus, IAV uses this mechanism to cause ZBP1/RIPK3-dependent necroptosis. MCMV murine cytomegalovirus, HSV herpes simplex virus, IAV Influenza A virus, vRNA viral RNA

ZBP1 was originally identified as a sensor of viral DNA that regulates virus-induced NF-κB activation and interferon expression [22, 23]. However, DNA-induced IFN expression was normal in *Zbp1*^−/−^ macrophages, dendritic cells and embryonic fibroblasts [24]. Hence, it is noteworthy that viral RNA sensing appears to be the key mechanism by which ZBP1 becomes activated in response to infection by mutant MCMV expressing a RHIM-mutated M45/vIRA. As we shall see below, viral RNA sensing by ZBP1 is also found in other viruses (Fig. 2). Hence, unlike the TNF/RIPK1/RIPK3 necroptosis pathway, which has a more prominent role in tissue homeostasis, ZBP1-mediated necroptosis functions predominantly as a critical host defense mechanism. These observations also argue strongly that the ZBP1/RIPK3 necroptosis pathway may be the product of host-pathogen co-evolution.

Besides M45, MCMV encodes another viral inhibitor called vICA (for viral inhibitor of caspase 8-induced apoptosis). vICA is encoded by the M36 gene. A recent study indicates that M36 and M45 cooperate with each other to suppress cell death-associated inflammation [18]. Infection of cells with mutant MCMV that lacks M36 and M45 led to caspase 8 and RIPK3/MLKL activation and severely reduced viral replication. The activation of RIPK3 and MLKL in the presence of intact caspase 8 activation is interesting and significant, since it challenges the paradigm that necroptosis induction requires caspase 8 inhibition. However, it is consistent with the involvement of ZBP1 rather than RIPK1 as the upstream activator of RIPK3 [19]. When compared to MCMV with single mutation in either M36 or M45, M36/M45 double mutant virus elicited stronger expression of many pro-inflammatory cytokines and early CD8^+^ T cell activation [18]. These results highlight the cooperative nature of apoptosis and necroptosis in driving robust host anti-viral inflammation.

Similar to MCMV, human cytomegalovirus (HCMV) also suppresses necroptosis induced by TNF, Smac mimetics and caspase inhibitors. However, in contrast to MCMV, HCMV does not inhibit RIPK3 phosphorylation, and MLKL phosphorylation was readily detected [25]. These results indicate that HCMV interferes with necroptosis at a step after MLKL activation. Consistent with the notion that HCMV does not target ZBP1 or RIPK3, super-infection with M45 RHIM mutant MCMV in HCMV-infected cells no longer sensitize the cells to necroptosis. While the precise mechanism by which HCMV inhibits necroptosis is still unknown, viral gene products under the control of the regulatory protein IE1 appear to be important, since infection with mutant HCMV lacking IE1 no longer conferred protection against TNF, Smac mimetic and caspase inhibitor-induced necroptosis [25] (Fig. 2). It will be interesting to determine if the differential mechanisms of necroptosis inhibition by MCMV and HCMV is the driver or consequence of viral evolution. It will also be important to evaluate how this switch in cell death inhibitory mechanism may influence anti-viral inflammation.

### Other herpesviruses

Similar to cytomegaloviruses, the alpha herpesviruses human herpes simplex virus 1 (HSV-1) and HSV-2 also modulate necroptosis through the large subunit of the ribonucleotide reductase (RR), ICP6 for HSV-1 and ICP10 for HSV-2. ICP6 and ICP10 share similar domain organization with M45, with an N-terminal RHIM and a C-terminal RR. However, in contrast to M45, ICP6 and ICP10 retain intact ribonucleotide reductase function. In addition, ICP6 and ICP10 interact with and inhibit caspase 8 [26]. Thus, they are dual inhibitors of apoptosis and necroptosis. The RHIM of ICP6 and ICP10 mediates RIPK1 and RIPK3 interaction [27–29]. HSV-1 triggered RIPK3/MLKL activation and necroptosis in mouse cells and increased viral titers and mortality in mice [28]. By contrast, HSV-1 or expression of ICP6 or ICP10 inhibits necroptosis in human cells. The differential effects of ICP6 and ICP10 in human versus mouse cells distinguish them from M45, which potently inhibits necroptosis in both human and mouse cells. Since HSV-1 and HSV-2 are human pathogens, the differential activity of ICP6 and ICP10 may account for their species tropism.

Epstein–Barr virus (EBV) is a gamma herpesvirus that infects the majority of human adults. A recent report suggests that the latent membrane protein 1 (LMP1) of EBV interacts with RIPK1 and RIPK3, modulates their ubiquitination status, and inhibits TNF, Smac mimetic and caspase inhibitor-induced necroptosis [30]. Interestingly, a mapping study using truncation mutants suggests that the interaction between LMP1 and RIPK1 or RIPK3 is independent of the RHIM. Further studies are required to confirm this observation and to examine whether this interaction plays any role in anti-viral immune responses and disease pathology.

### Viral FLIPs and necroptosis

Like caspase 8, cellular FLICE (caspase 8)-like inhibitor protein (cFLIP) contains two tandem death effector domains at the N-terminus. However, the long form of cFLIP (cFLIP_L_) harbors mutations at the C-terminal caspase domain, thus rendering it enzymatically inactive. By contrast, the short form of cFLIP, cFLIPs, lacks the C-terminal caspase domain. While cFLIP_L_ promotes caspase 8 activation through heterodimerization, cFLIPs inhibits caspase 8 activity [31]. Interestingly, a class of viral FLIP proteins (vFLIPs) has been identified in herpesviruses and the human poxvirus *Molluscum contagiosum virus* (MCV) [32, 33]. vFLIPs lack the C-terminal caspase domain and thus resemble cFLIPs in both structure and function [34, 35]. Surprisingly, many vFLIPs also exhibit necroptosis inhibitory activity [9]. vFLIPs have been reported to interact with RIPK1 [36]. However, we were not able to detect such interaction (unpublished observation). Hence, the mechanism by which vFLIPs inhibit necroptosis remains elusive.

Besides apoptosis and necroptosis, vFLIPs also interfere with NF-κB activation. Several reports showed that transgenic expression of MC159 inhibited NF-κB activation and triggered a loss of memory CD8^+^ T cells in mice [37, 38]. However, NF-κB inhibition requires high expression of MC159. At low level of expression, MC159 actually stimulates NF-κB activation [39]. In contrast to transgenic mice expressing high level of MC159, mice with low MC159 expression exhibited enhanced NF-κB activation, chemokine expression and viral clearance in response to VV infection [39]. The functional relevance of MC159 in virus infection was further characterized by Hüttmann and coworkers. When expressed in recombinant MCMV lacking either M36 or M45, MC159 blocked TNF-induced apoptosis. However, necroptosis in human, but not mouse cells, was inhibited by recombinant MCMV expressing MC159 [40]. These discrepant results highlight a potential caveat in using over-expression to study viral necroptosis inhibitors suggesting that their behavior can vary depending on the expression level and the cell type studied.

### Necroptosis in RNA virus infection

In a survey for additional viruses that might interfere with necroptosis, Schock and colleagues found that Sendai virus induced necroptosis in the presence of the pan-caspase inhibitor zVAD-fmk [41]. Sendai virus drives necroptosis via the RNA sensor RIG-I [41]. In *Ripk3*^−/−^ mice, Sendai virus infection resulted in increased inflammation. Although more work is required to fully elucidate the mechanism by which Sendai virus triggers necroptosis and inhibits inflammation, these results do highlight the fact that RIPK3 does not always facilitate detrimental inflammation. Rather, in certain situations such as that in Sendai virus infection, RIPK3 can play an anti-inflammatory and protective role.

Recently, necroptosis has emerged as a critical pathway in influenza virus-induced cell death and pathology. For instance, Rodrigue-Gervais and coworkers found that *Birc3*^−/−^ mice, which are deficient in the anti-apoptotic factor cIAP2, were hypersensitive to influenza A virus (IAV) infection and developed severe loss of lung epithelium [42]. Importantly, pharmacologic inhibition of RIPK1 kinase activity or genetic deletion of *Ripk3* rescued *Birc3*^−/−^ mice from IAV-induced epithelial necrosis, tissue damage and lethality. These results are consistent with the known function of cIAP1 and cIAP2 as apoptosis and necroptosis inhibitors.

The study in *Birc3*^−/−^ mice suggests a role for RIPK1 and RIPK3 in IAV pathogenesis. Indeed, several recent studies indicate that RIPK3-dependent necroptosis plays a key role in IAV-induced cell death. Similar to infection with MCMV, ZBP1 functions as the upstream sensor of IAV RNA to activate RIPK3 [43, 44]. ZBP1 was reported to sense IAV nucleoprotein (NP) and polymerase subunit (PB1) to induce necroptosis [43]. Interestingly, NP was also reported to induce apoptosis in IAV-infected cells [45, 46]. In contrast to *Birc3*^−/−^ mice, RIPK1 was mostly dispensable for IAV-induced cell death in wild type cells or mice [47]. *Ripk3*^−/−^ and *Zbp1*^−/−^ mice failed to control viral replication and exhibited increased mortality in response to IAV infection [44, 48]. However, increased mortality was not observed in another report [43].

Mechanistically, IAV-induced expression of cytokines such as type I IFNs via RIPK3 and ZBP1. As such, IAV-induced IFNβ expression was impaired in *Ripk3*^−/−^ and *Zbp1*^−/−^ cells [43]. While reduced IFNβ expression might be due to decreased necroptosis and necroptosis-driven inflammation, it could also be caused by direct effects on gene transcription [49–53]. For example, in West Nile virus infection, chemokine expression in the central nervous system was driven by RIPK3 but independent of cell death [52]. In macrophages, IAV induced binding between RIPK3 and the mitochondrial associated RNA sensor MAVS, which stimulated TBK1 and IRF1/IRF3 activation to promote IFNβ mRNA stability [48, 54]. This response is not only critical for virus-induced IFN expression, but also for the expression of the necroptosis genes *Zbp1* and *Mlkl*. The relative contribution of cell death-dependent and independent activities of RIPK3 in influenza virus infection will require further investigation.

Further evidence supporting a role for RIPK3-dependent necroptosis in shaping influenza virus-induced pathogenesis comes from a recent study comparing the host response to seasonal versus pandemic strains of IAV. Remarkably, virus-induced necroptosis in dendritic cells (DCs) was readily detected by seasonal IAV strains, but not by pandemic IAV strains [55]. In co-culture experiments, seasonal IAV-infected DCs underwent necroptosis and induced bystander DC maturation and virus-specific T cell proliferation. By contrast, DC maturation and T cell proliferation were significantly impaired in pandemic IAV-infected DC cultures. At present, the molecular mechanism by which seasonal and pandemic IAV strains induces differential necroptosis in DCs is unknown. However, differences in the viral hemagglutinin gene segment between the two groups of IAVs have been implicated [55]. These results highlight the interesting scenario that necroptosis may have broad functions in shaping viral evolution and pathogenicity.

To further complicate matters, recent studies have clearly demonstrated a necroptosis-independent role for RIPK3 inNLRP3 inflammasome activation [56]. Inflammasome activation results in two major biological outcomes: processing and secretion of pro-inflammatory cytokines IL-1β and IL-18, and induction of pyroptosis, another lytic form of cell death [57]. RIPK3-mediated inflammasome activity has been reported to promote IL-1β secretion in response to IAV, Sendai virus and Vesicular stomatitis virus (VSV). Interestingly, the mitochondrial fission protein DRP1 was implicated to signal downstream of RIPK3 to promote RNA virus-induced inflammasome activity [58]. However, these results were challenged by another study, which showed that VSV-induced IL-1β secretion was normal in *Ripk3*^−/−^macrophages and macrophages expressing the kinase inactive RIPK3 mutant K51A [59]. Nonetheless, the artificial ligand for TLR3 and RIG-I poly(I:C), which mimics viral double-stranded RNA, could clearly activate RIPK3 signaling, especially in the presence of caspase inhibition [60–62]. Thus, we cannot rule out the possibility that RIPK3 can indeed stimulate inflammasome activation in certain situations. In this light, it is noteworthy that RIPK3-dependent inflammasome activity was particularly prominent in human monocytes, mouse dendritic cells and macrophages [49, 63, 64]. Thus, the anti-viral mechanism employed by RIPK3 in response to different pathogens will likely vary depending on the cell type under investigation.

## Conclusion

Research in the last decade has clearly established necroptosis as a crucial host defense response against many pathogens. In response, viruses have developed different strategies to neutralize the host necroptosis machinery. In addition to viral pathogens, an increasing number of non-viral pathogens have also been found to target the necroptosis machinery. For example, atypical proteases from certain pathogenic bacteria specifically target mammalian RHIM-containing signal adaptors for cleavage and inactivation [65]. An important lesson we learned from studies with different pathogens is that while apoptosis and necroptosis are often mutually exclusive responses in tissue culture, this is not the case in pathogen infections. For instance, IAV infection elicits concomitant activation of caspase-dependent apoptosis and RIP kinase-mediated necroptosis [66]. The cooperative nature of apoptosis and necroptosis in anti-microbial defense is further illustrated by the fact that caspase 8, its adaptor FADD, RIPK1 and RIPK3 are often found in the same signaling complex regardless of whether apoptosis or necroptosis is the dominant response. This concept is further bolstered by the many examples in which pathogens often target both pathways of cell death. The knowledge gained from studying how various pathogens counteract the host cell death machinery may lead us to better therapeutic strategies against viral and sterile inflammatory diseases.
